# What Is Lost in the Weismann Barrier?

**DOI:** 10.3390/jdb8040035

**Published:** 2020-12-16

**Authors:** Abigail P. Bline, Anne Le Goff, Patrick Allard

**Affiliations:** 1 Molecular Toxicology Interdepartmental Program, University of California Los Angeles, Los Angeles, CA 90095, USA; abigailbline0@ucla.edu; 2 UCLA EpiCenter on Epigenetics, Reproduction & Society, University of California Los Angeles, Los Angeles, CA 90095, USA; alegoff@ucla.edu; 3 Institute for Society & Genetics, University of California Los Angeles, Los Angeles, CA 90095, USA

**Keywords:** Weismann barrier, germline, germ cells, soma, transgenerational epigenetic inheritance

## Abstract

The Weismann barrier has long been regarded as a basic tenet of biology. However, upon close examination of its historical origins and August Weismann’s own writings, questions arise as to whether such a status is warranted. As scientific research has advanced, the persistence of the concept of the barrier has left us with the same dichotomies Weismann contended with over 100 years ago: germ or soma, gene or environment, hard or soft inheritance. These dichotomies distract from the more important questions we need to address going forward. In this review, we will examine the theories that have shaped Weismann’s thinking, how the concept of the Weismann barrier emerged, and the limitations that it carries. We will contrast the principles underlying the barrier with recent and less recent findings in developmental biology and transgenerational epigenetic inheritance that have profoundly eroded the oppositional view of germline vs. soma. Discarding the barrier allows us to examine the interactive processes and their response to environmental context that generate germ cells in the first place, determine the entirety of what is inherited through them, and set the trajectory for the health status of the progeny they bear.

## 1. Introduction

The idea of the Weismann barrier provides a conceptual framework for the relationship between germ cells and somatic cells and the larger extraorganismal environment. The considerable influence of this framework is, perhaps unsurprisingly, matched by manifold ways of understanding it depending on the time, the writer and the scientific area. The basic premise of the Weismann barrier, that is shared across its varied uses, is that germ cells are fundamentally separate from somatic cells. The larger implications of the barrier concept bear on the nature of germ cells, the mechanisms of inheritance, and the course of evolution.

The Weismann barrier is typically applied as a self-evident label and rarely defined, suggesting that it rests on an uncontroversial piece of evidence [1,2]. Taking a historical view on the origins of the Weismann barrier shows that it is hardly the case. August Weismann was trained as a medical practitioner and chemist in the late 1800s prior to conducting research in zoology and embryology [3]. In a pre-genetics era, he worked to understand how species can be relatively stable in their characteristics across generations, yet exhibit enough variation for substantial change to occur over evolutionary time in accordance with Charles Darwin’s theory of natural selection. Weismann’s solution to this problem was described in his seminal 1893 work *The Germ Plasm: A Theory of Heredity*, in which he proposed a nuclear substance called the “germ plasm” as the essential unit of inheritance. The germ plasm was the theoretical foundation upon which Weismann built his inferences.

Although Weismann’s model of the germ plasm was never widely accepted, the concept of a barrier named for him has been widely used as a default framework in which new scientific evidence and theories must be constructed. Modern-day scientific articles still refer to “crossing the Weismann barrier” [1,4] and evidence undermining the existence of such a barrier is described in this framework as an exception to the rule or a “leak” in the barrier. In this article, we seek to reevaluate the framework of the barrier itself and its limitations in light of contemporary scientific knowledge and to explore how scientific pursuits might advance differently without the framework of the Weismann barrier.

## 2. Weismann’s Model of Inheritance

With his germ plasm theory, Weismann sought to explain how species transform over evolutionary time depending on their environment yet preserve relative constancy between each generation. To understand this theory, it is necessary to understand the hypothetical physical organization and evolutionary origins of Weismann’s unit of inheritance: the germ plasm. According to Weismann, the germ plasm itself was an assembly of three “vital units”, that is, a unit exhibiting the primary “vital forces” of assimilation and metabolism, growth, and multiplication by fission. The smallest unit was the “biophor” [5]. Biophors were made up of varying types, numbers, and arrangements of molecules that could occur in unlimited combinations. The particular composition of a given biophor determined the structure that it formed in the cell and Weismann considered the biophors the “bearers of the cell qualities” [5]. Biophors could only be produced from pre-existing biophors and bore “historical” qualities formed over time through heredity and selection.

Weismann thought that in the earliest unicellular organisms, the biophors were freely dispersed throughout the cell [5]. In this scenario, changes induced to a biophor in any part of the cell could be inherited with the next cell division by binary fission. However, with the evolution of the nucleus, the heritable biophors capable of reproducing the entirety of the organism were sequestered to this structure alone. The separation of the heritable material into the nucleus also meant that external influences that acted upon and changed the cell body could no longer be transmitted to offspring; only changes to the nuclear substance were heritable. It appears that Weismann believed the nucleus provided a degree of protection that made the nuclear contents less susceptible to external influences and thus more stable over time. Although different biophors could grow and reproduce unevenly in response to factors such as nutrient availability and temperature due to their different physicochemical properties, Weismann thought that substantial transformations of the biophors did not occur easily or rapidly. Thus, the relative constancy of the nuclear substance between generations provided a defining feature of a particular phylogenetic lineage.

**Figure 1:**
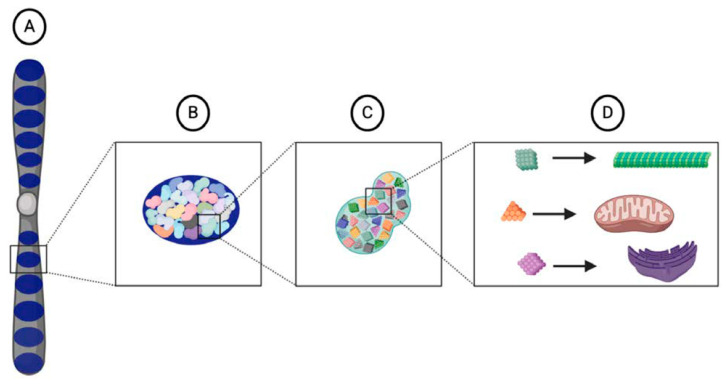
Illustration of Weismann’s vital units. Each chromosome, or idant (**A**), is composed of several ids (dark blue) (**B**) that are inherited intact from the parental germ cells within the germ plasm. Each id is composed of determinants (**C**) that are arranged in a particular architecture. Each determinant corresponds to a specific cell type in an organism and contains a particular assemblage of biophors (**D**), a group of molecules that, when released from the nucleus and into the cytoplasm, provide the starting material from which cellular components develop.

As organisms became more complex and evolved different cell types, Weismann reasoned that the organization of the nuclear substance also became more complex. While different biophors corresponded to different structures in a cell, they did not correspond directly to a particular cell type [5]. Instead, different combinations of biophors were organized into a higher-order vital unit called a “determinant” (Figure 1). As the name implies, Weismann thought that determinants conditioned the character of a cell; one type of determinant might correspond to a muscle cell while another determinant might correspond to a skin cell. In addition to organizing the biophors, determinants also served to control their activity. Within the structure of a determinant, biophors were maintained in an “inactive” state where they could only grow and reproduce. However, once released from a determinant, biophors were small enough to move through the nuclear pores and into the cytoplasm, where they were physically used up to produce the structures of the cell. This regulatory power over the biophors’ activity provided the nuclear contents with the ability to control “the body of the cell” [5].

The determinants were organized into the highest-order vital unit called the “id.” Several ids were aligned along the length of each chromosome (Figure 1), which Weismann in turn called an “idant” [5]. In sexually reproducing organisms, half of a zygote’s ids originated from the mother while the other half originated from the father. Weismann thought that the different combinations of ids inherited from the parents was one of the main sources of variation in a species. Although all the ids of a mature gamete were very similar, they were not identical and had very slight differences in their precise organization and composition. These differences occurred in part due to the different ancestral lineage of each id. Weismann thought that the “reducing divisions” of meiosis randomly determined which ids were retained in a gamete. Therefore, even though a zygote received half of its ids from each parent, the share of ids inherited from each grandparent could not be predicted. Because each id had its own heritage and contained all the determinants necessary to reproduce an organism, Weismann alternatively called an id the “ancestral germ plasm” [5].

**Figure 2:**
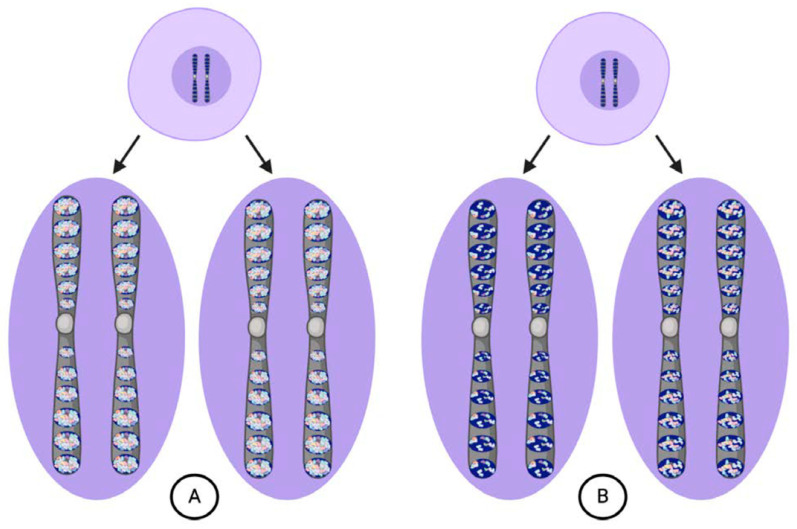
According to Weismann, cells can undergo either ordinary (**A**) or embryogenic divisions (**B**). (**A**) Ordinary divisions produce daughter cells with chromosomes bearing vital unit composition identical to the parental cell and maintain the germ plasm intact from the zygote through mature germ cells. (**B**) Embryogenic divisions produce daughter cells with chromosomes that bear different determinants in their ids depending upon their destined cell lineage.

All of the ids together constituted the germ plasm proper, which Weismann argued was the sole heritable substance transmitted to each new generation via the germ cells [5]. Similar to the three vital units composing it, Weismann thought that the germ plasm could only be propagated from a pre-existing germ plasm. Most importantly, what was transmitted in the germ plasm was its “fixed architecture”, or the particular arrangements and proportions of the different vital units [5]. The architecture of the germ plasm was essential because it determined the entire course of ontogeny. While evolutionary time built up the structure of the germ plasm, it was deconstructed in an individual over developmental time. Weismann referred to this deconstruction process as “blastogenesis”, or origin from a germ plasm. From the zygote, Weismann thought that cells could replicate through “ordinary” cell divisions, where exact copies of the nuclear contents are replicated in the daughter cells, or “embryogenic” cell divisions, where daughter cells receive unequal shares of the nuclear contents (Figure 2). Ordinary divisions producing exact copies of the complete germ plasm created a “reserve” that was maintained intact in cells of the germ lineage. Embryogenic divisions generated somatic “idioplasm” that retained only part of the germ plasm. The original architecture of the germ plasm dictated the order in which the idioplasmic ids were deconstructed and their determinants distributed into daughter cells.

Thus, the germ plasm had a dual role in inheritance and development. The ability to reproduce an organism as a whole was held only by cells with reserve germ plasm that was unchanged from the unification of the parental gametes in the zygote. Once the germ plasm was broken up, the resulting idioplasm only had the potential to generate cell types corresponding to the types of determinants the idioplasm retained, but not the entire organism [5]. Any perturbations that caused changes in the idioplasm could cause variations in cells produced from that idioplasm’s lineage. However, according to Weismann, idioplasm could never be reconstructed into germ plasm and such “somatogenic” variations could not be transmitted to subsequent generations via the germ plasm. Thus, any changes that arose in an organism’s body during development were independent from what would be transmitted to progeny. Only direct changes to the composition of the reserve germ plasm were heritable. Such changes might result from changes in growth rates of the vital units due to differential effects of nutrition or temperature on the different molecular components. However, Weismann argued that such changes would be imperceptibly small within a single generation.

Weismann explicitly framed his formalized theory of the germ plasm as a rebuttal to Charles Darwin’s pangenesis hypothesis [5] and a general rejection of the idea of inheritance of acquired characteristics (IAC). Jean-Baptiste de Lamarck’s theory of IAC, where phenotypic changes arising from patterns of use or disuse in an organism’s body are transmissible to their offspring was a widely held view throughout the 19th century and informed Darwin’s thinking [6]. According to the pangenesis hypothesis, all cells continually release self-replicating units called “gemmules” that freely circulate throughout the body, but concentrate in the sexual organs [7]. Darwin proposed that pangenesis was the mechanism that generated heritable variation upon which natural selection could act. While Weismann was an early supporter of Darwin’s idea of natural selection and also initially accepted IAC [8], his model of the germ plasm rendered any somatic influence on inheritance impossible.

## 3. Origins of the Weismann Barrier

The inability of the parental body to influence the characteristics of the offspring and the direct refutation of IAC was a key implication and the main thesis that later scientists retained from Weismann’s work [9]. However, while Weismann’s argument against IAC was firmly derived from the hypothetical structure and function of the germ plasm, the theory of the germ plasm itself was almost universally rejected by other scientists and is largely inconsistent with contemporary understanding of biology. Weismann was most widely criticized for the speculative nature of his theory and the lack of clear experimental or cytological evidence supporting it [10]. He himself acknowledged that it was not possible to discern the structure of the germ plasm with available technology and that the structure he proposed was based on deductive reasoning. Consequently, scientists who wanted to accept Weismann’s conclusion against IAC provided reinterpretations that maintained the separation of the body from inheritance while eliminating the germ plasm as a factor.

In the earliest and most basic reinterpretation of Weismann’s theory, the cell biologist Edmund Beecher Wilson [11] replaced the germ plasm with the germ cell. According to Wilson, the germ cells, through division, give rise to both soma and new germ cells, which separate from the soma and repeat the process in each successive generation [11]. It was the germ cell that offspring inherited from the parents, not their body, and the characteristics from the germ cell were retained independent from the body via the line of intergenerational germ cell descent. In his influential textbook *The Cell in Development and Inheritance*, Wilson represented this idea with a diagram that is now widely attributed to Weismann [12]. The diagram depicts the line of inheritance running from the parental germ cells through the germ cells of each new generation, with the somatic cells of each individual branching away from the germ cells and outside the realm of inheritance. This diagram ostensibly representing Weismann’s idea was adopted and refined, in particular in another influential biology textbook written by Simpson et al. in 1957. In this text, the authors shifted the argument for the separation of germ cells from the somatic cells: they reasoned that “undifferentiated” germ cells could not be derived from differentiated cells of the body but instead must come directly from the lineage of undifferentiated cells that remain such across generations [13]. In both Wilson’s and Simpson et al.’s books, the perpetual separation of the germ cells from the somatic cells, as well as the exclusive generation of the latter from the former, made IAC impossible.

An alternative means of reinterpreting Weismann’s theory to reject IAC was the replacement of the germ plasm with the genotype [10]. Weismann’s notion that hereditary units contained within the gametes of the parents were causal in determining the physical qualities of the resulting offspring, without being determined by the qualities of the parents themselves, was bolstered by the rediscovery of Gregor Mendel’s 1866 work on heredity in 1900 [14,15]. Weismann himself was unaware of Mendel’s work when he was developing the germ plasm theory in the 1880s and 1890s. This rediscovery led to the emergence of genetics as a distinct field of science. In a parallel way to Weismann, many early geneticists emphasized the idea of continuity of the hereditary substance between generations, and that this hereditary substance was borne by the chromosomes [14]. Weismann was also credited with helping to initiate the “genotype concept”, in which the totality of the inherited genes determines an organism’s phenotype but the phenotype has no bearing on what is inherited [10]. The unification of neo-Darwinian evolution and Mendelian genetics with the modern synthesis of the 1930s and 1940s [16] further promoted the idea that a genetic program selected over evolutionary time ultimately determined phenotype [17]. Evidence of control of the phenotype by an inherited genetic program was seen by some as a “final blow” to IAC.

Aside from bolstering the rejection of IAC, these reinterpretations of Weismann’s work converge on a notion of unidirectionality. Cellular transformations and information conveyance can proceed in a one-way direction only, in effect creating a barrier to reversion. In a developmental version of the barrier, somatic cells differentiated from parental gametes can neither revert to germ cells [4] nor influence what is inherited via the germ cell lineage [18]. In a genetic version of the barrier, the phenotype cannot recreate the genetic information from which it was derived [19], nor can it influence the heritable genetic information conveyed across generations [20]. Although these ideas diverge from Weismann’s original germ plasm theory to varying degrees, they have all been used to define the term “Weismann barrier.” While the Weismann barrier presents a relatively simple framework for development and inheritance, it is questionable as to whether this framework is particularly useful going forward.

## 4. What Is Lost in the Weismann Barrier?

**Figure 3:**
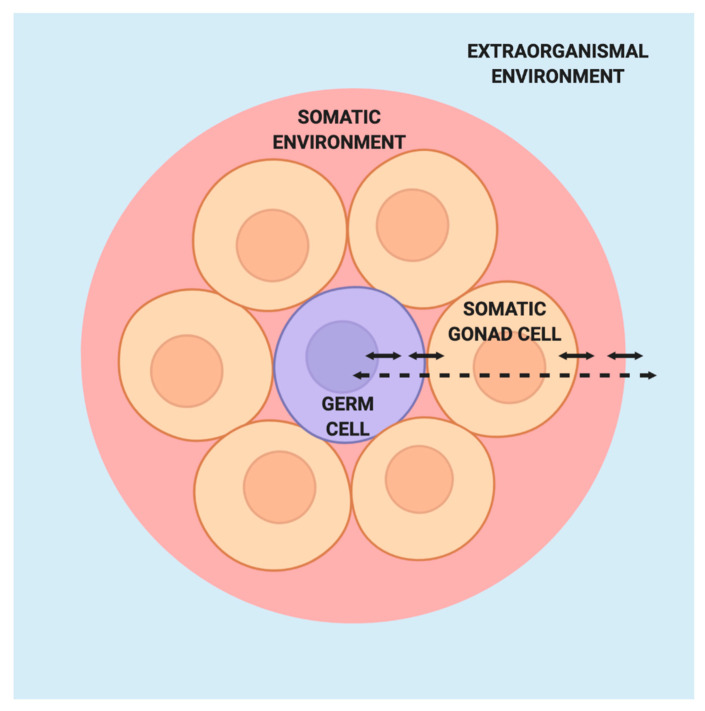
Model of germ cells in the absence of the Weismann barrier. Within the germ cell (purple), interactions between the nuclear and cytoplasmic components integrate signals from the surrounding somatic gonad cells, which themselves are responsive to signals from the germ cell. The germ cells and somatic gonad cells are situated within a larger somatic environment that is in turn influenced by the extraorganismal environment.

The barrier concept presents germ cells and the inherited phenotype as fixed states while offering no explanation for the processes by which these states came to be. To some extent, it implies that no such explanation is necessary, for the inherent properties of germ cells and the genome alone appear sufficient to determine the course of development and inheritance. However, the barrier concept stands in stark contrast to what we know about the myriad processes regulating germ cell fate and function as well as their interactions with surrounding somatic cells and the external environment (Figure 3). The regulatory processes and interactions that the barrier concept relegates to obscurity are precisely what need to be elucidated to further our understanding of germ cell biology, inheritance, and human health.

### 4.1. Emergent Germ Cell Fate and Function through Interaction with Somatic Cells

Rather than being perpetually independent from somatic cells, germ cells across many different species are intimately linked with somatic cells throughout development. In mammals, primordial germ cells (PGCs) are induced by a combination of bone morphogenic proteins (BMPs), Wnt proteins, and other signaling molecules provided by the surrounding embryonic tissues [21]. During migration from the site of induction to the gonadal ridge, PGC chromatin is dramatically remodeled, exhibiting a dramatic loss in DNA methylation and an increase in repressive histone marks [22]. It is thought that this epigenomic reprogramming is necessary for PGCs to attain germ cell competence. While the precise mechanisms regulating these epigenomic changes, are not fully known, in vitro models of primordial germ cell-like cells (PGCLCs) suggest that these changes are at least in part due to interactions with somatic cells. PGCLCs can be specified from embryonic stem cells (ESCs) or induced pluripotent stem cells (iPSCs) derived from somatic tissues [23] using condition media to drive expression of germ cell-specific transcription factors. However, once specified, PGCLCs do not substantially proliferate, survive for extended periods of time, or progress through epigenomic programming without using somatic feeder cells in the culture [24,25]. Furthermore, PGCLCs cannot develop further and ultimately produce functional gametes unless they are either co-cultured with somatic gonad cells [26] or transplanted into a gonad in vivo [24].

Across species, the somatic gonad is necessary for regulating meiosis [27,28] and supporting germ cell function through later development and maturation. Within the gonad, germ cells and somatic cells directly communicate with one another through gap junction connections [29,30,31,32,33], endocytosis [34], and extracellular vesicles [35]. Through these means, both male and female germ cells receive nutrients, small non-coding RNAs (sncRNAs), proteins, metabolites, hormones, and other signaling molecules from the surrounding somatic cells. Somatic cell interactions with germ cells appear to be well conserved from invertebrates to mammals, suggesting their importance in germ cell function. In *Caenorhabditis elegans*, mutations in the innexin proteins that form the gap junctions between germ cells and the somatic gonad result in loss of germ cell proliferation, embryonic lethality, and sterility [32]. Laser ablation of gonad sheath cells results in failure of oocytes to progress through meiosis I, leading to infertility [36]. In both male and female mice, knockout of the connexins forming the gap junctions between germ cells and somatic cells impedes meiotic progression and germ cell maturation [29,31]. The inability of aged granulosa cells to efficiently generate new gap junctions with oocytes may contribute to increasing infertility with maternal age [30]. In male mammals, extracellular vesicles called epididymosomes deliver somatically-derived sncRNAs to maturing spermatozoa and are necessary to support pre-implantation embryonic development [37,38]. Thus, available evidence indicates that somatic cells are not strictly separated from germ cells but essential to germ cell maturation and reproductive competence.

### 4.2. A Broader View of Heritable Information

Although the barrier concept indicates that the inherited genome sequence alone is sufficient to directly determine phenotype, a substantial degree of phenotypic variation can be generated through layered regulatory processes that control how, when, and where the genome may actually be expressed. From the start of metazoan development, the single-celled zygote differentiates into a wide range of cell types from a single genome. It is not genes per se but the multitudinous ways in which they can be modified, activated with others, and/or silenced that allow organisms to change throughout their life course and in response to a changing environment. The flexibility of genome expression is necessary for the act of living as a multicellular organism and for perpetuating life through reproduction. Increasing evidence indicates that factors inherited from parental germ cells via influences from the somatic cells and/or the larger environment can modulate genome expression and alter phenotype in ways comparable to a change in genetic sequence.

The direct influence of heritable information flow from soma to the germline is particularly well understood in rodents. A recent and rapidly growing body of work has shown that the sncRNA content of sperm, particularly tRNA fragments, is highly influenceable by environmental changes such as diet and stress [39]. As shown by zygote microinjection experiments in mice, such modified tRNA fragments extracted from sperm are sufficient to modify the offspring’s phenotypes, including metabolic dysfunction in the case of paternal high-fat diet [40]. Other types of sncRNA and long non-coding RNAs (lncRNA) can also convey heritable information that alters the phenotype of offspring whose fathers were subjected to chronic stress or trauma [41,42]. Based on available evidence, it appears that alterations in sperm sncRNA may be attributable at least in part to the composition of epididymosomes taken up by the sperm from the epididymal cells. As noted previously, the contents of epididymosomes are necessary for sperm to support the regulation of early embryonic development and embryonic viability [37,43]. Therefore, environmental or other influences that alter the contents of the epididymosomes and thereby the paternal germ cells may have a direct impact on the phenotype of their offspring.

Increasing evidence indicates that environmental influences on non-genetic heritable information can alter offspring phenotype for more than one generation. The last few years have seen a rapid expansion of the field of transgenerational epigenetic inheritance (TEI) in a wide variety of animal models, including *C. elegans*, zebrafish, *Drosophila*, and mouse [44]. Work in the nematode has demonstrated that the impact of numerous environmental cues can be inherited for several generations and provided a molecular understanding of the mechanisms of soma-to-germline and germline-to-germline communication that direct TEI. A recent study demonstrated that in *C. elegans*, small RNAs synthesized in neurons through the RDE-4 pathway alter the expression of 124 mRNAs and 1287 sncRNAs and trigger transgenerational changes in the offspring’s transcriptome and chemotaxis behavior [45]. How the neuronal RDE-4 derived sncRNAs impact the germline transcriptome is not fully elucidated but appears to be largely independent of the activity of the siRNA membrane transporter SID-1 [45,46]. It is possible that a non-SID-1 mechanism of transport of sncRNAs or a relay mechanism involving other signaling molecules such as hormones may be at play. This work highlights the ability for factors that are uniquely produced by neurons to impact the germline and the phenotype of the resulting progeny.

A recent study in mice suggests another mechanism of TEI may involve changes to the genome’s physical structure [47]. The eukaryotic genome is non-randomly arranged within the nucleus and, at the highest level, is organized into spatially segregated heterochromatin and euchromatin compartments [48]. Although partly influenced by DNA base pair sequence [47], chromatin compartmentalization may largely be driven by phase separation [49]. Phase separation of chromatin is a physicochemical process dependent upon factors affecting intra-/intermolecular forces, such as solute concentration and temperature, as well as features of the genome affecting charge and binding affinity, such as histone post-translational modifications [50]. The local chromatin environment within the phase boundary concentrates proteins and other biomolecules that in turn can affect transcription and reinforce or alter chromatin compartmentalization by modifying DNA and histones [51]. It appears that perturbations to chromatin compartmentalization in response to tributyltin (TBT) exposure during early embryonic development could shift genomic regulatory circuits and impact metabolic phenotype [47]. Furthermore, the authors hypothesize that the resulting changes expression of chromatin organization-related genes could be carried through the adult’s germ cells and be “reconstructed” to repeat the cycle in the next generation. These effects appeared to persist in the fourth-generation male offspring. Although changes in DNA methylation patterns were also observed in this generation of offspring ancestrally exposed to TBT, these changes did not correlate with patterns of mRNA expression. These data suggest that the three-dimensional organization of the genome, in addition to sequence, may in effect be heritable across generations.

The debate regarding what information is heritable between generations has mainly been framed in terms of evolutionary relevance. Over evolutionary time, only information that may be faithfully replicated in perpetuity is generally considered to be truly heritable, and thus relevant [19]. However, at the scale of the human lifespan, the perpetual transmissibility of non-genetic heritable information, or lack thereof, becomes less relevant. As long as the phenotype, or more importantly the health status, of at least one generation can be negatively affected by environmentally-induced changes in heritable information transmitted via the germline, it warrants further study to better understand the mechanisms by which this happens and pursue interventions that may improve human health.

## 5. Conclusions

The writings of August Weismann over 100 years ago helped instill a modern concept of a barrier according to which the germ cells and their genome function independently from the body of an organism. Remarkably, Weismann himself acknowledged that his germ plasm theory, which led to the barrier concept, was a product of deductions from available information and not a proven fact: “It is nevertheless possible that continuity of the germ plasm does not exist in the manner in which I imagine that it takes place, for no one can at present decide whether all the ascertained facts agree with and can be explained by it. Moreover, the ceaseless activity of research brings to light new facts every day, and I am far from maintaining that my theory may not be disproved by some of these. But even if it should have to be abandoned at a later period, it seems to me that, at the present time, it is a necessary stage in the advancement of our knowledge, and one which must be brought forward and passed through, whether it prove right or wrong, in the future” [52]. We now know that it is not the contents of the chromosomes that differs between germ and somatic cells but rather the manner in which they regulate and express the genome. The barrier concept has gained so much prominence over the 20th century because it facilitated scientific progress in some respects, in particular genetic and evolutionary understandings of inheritance. Without dismissing the historical contributions Weismann made to the advancement of knowledge, it may be time to accept that utilization of the barrier concept is a stage through which we have fully passed.

Continued adherence to the barrier concept is extremely limiting in furthering our understanding of germ cell biology and inheritance. Germ cells and their genomes are not abstract conveyors of information but physical structures that can change form and function in numerous ways in response to a combination of cell-autonomous and non-autonomous cues. It is this ability to change as a part of an integrated organismal system that determines both physiological and pathophysiological reproductive outcomes in an individual. The capacity to reproduce is itself a phenotype facilitated by concerted functions and inter-relationships between germ and somatic cells and the germ cell genome is not entirely separable from the larger context of the body in which it is located. Whereas the barrier concept takes as a starting point the germ cells and the resulting phenotype produced through inheritance, discarding the barrier allows us to unboundedly examine the interactive processes and their response to environmental context that generate germ cells in the first place, determine the entirety of what is inherited through them, and set the trajectory for the health status of the progeny they bear.
